# Dr. A. B. Straight

**Published:** 1912-02

**Authors:** 


DR. A. B. STRAIGHT of Hornell, died January 9, 1912, aged 46. He was formerly a Methodist minister. He was a graduate in medicine of the P. & S. of Baltimore, 1891.

